# Adolescent Interview With a Medical Interpreter: A Standardized Patient Encounter for Pediatric Residents

**DOI:** 10.7759/cureus.47279

**Published:** 2023-10-18

**Authors:** Tristin Hudson, Shaina M Hecht, Cynthia Robbins, Megan S McHenry, Bobbi Byrne

**Affiliations:** 1 Department of Pediatrics, Indiana University School of Medicine, Indianapolis, USA

**Keywords:** confidentiality, medical interpreter, adolescent medicine, simulation in medical education, pediatrics, standardized patient

## Abstract

Background: Pediatricians can cultivate a more trusting relationship with their non-English speaking patients by emphasizing the importance of upholding patient confidentiality in the presence of an interpreter. We designed a case for pediatric residents to increase comfort when conducting an adolescent interview using a medical interpreter, emphasizing the importance of upholding patient confidentiality, specifically when discussing sensitive health topics.

Methods: We developed a standardized patient encounter (SPE) for pediatric residents at a large academic institution that focused on exploring sensitive health topics with an adolescent, non-English speaking female patient using an interpreter. A validated survey was administered upon completion of the case, prompting participants to reflect on their comfort and skills with specific activities before and after the case, and was analyzed using paired t-tests.

Results: Eighty-nine residents participated; 66 were interns and 23 were in their second year of residency. The mean scores of all paired survey items significantly increased after the case (*p*<0.01), indicating perceived personal growth in all educational objective categories. The majority of the participants (97%, N=86) agreed that they built skills in understanding cultural humility and caring for future patients (mean Likert scores: 4.91 and 5.10, respectively).

Conclusions: Upon completion of the case, residents reported increased comfort and skills using a medical interpreter for non-English speaking patients within the context of patient-centered care, discussing various sensitive health topics, emphasizing the importance of upholding patient confidentiality, and demonstrating skills in adolescent interviewing. Both medical confidentiality and cultural humility education can be integrated into simulation-based medical education to improve the quality of care for diverse patient populations.

## Introduction

Communication is a core foundation of a patient-provider relationship, and the delicate complexities of this relationship can be compromised when an interpreter is introduced to the situation [1]. Especially when using an interpreter, physicians must ensure the establishment of trusting relationships with their non-English speaking patients, allowing them to feel comfortable sharing concerns about their health and seeking professional advice [2]. Although medical interpreters are bound by a code of ethics to uphold confidentiality, a primary concern of patients who need to use a medical interpreter is the perceived lack of confidentiality, which can naturally lead to a lack of trust from the patient [3]. There is evidence that non-English speaking patients have lower patient satisfaction and poorer health outcomes [4].

These challenges can be further exacerbated when discussing sensitive topics, especially in populations with emerging autonomy, such as adolescents. Within the standard adolescent interview, the HEEADSSS exam is often used to document the social history and screen critical preventative health topics for adolescents [5]. The respective letters of the HEEADSSS acronym describe various topics to discuss during the interview: home, education/employment, eating, activities, drugs, sexuality, suicidality, and safety [5]. It is important for physicians to ask open-ended questions regarding these topics in order to provide an opportunity for patients to clearly and fully explain their concerns [6]. While adolescent patients are building skills in managing their own medical care, they may feel hesitant to discuss more sensitive health issues if rapport and trust are not established [7]. Upholding confidentiality is a key aspect of building trust in patient-provider relationships, especially those involving adolescents [7]. 

Practicing cultural humility is a strategy that medical providers can use to overcome these challenges, especially for providers who differ culturally from their patient population. Cultural humility has been defined as “a lifelong commitment to self-evaluation and critique, to redressing power imbalances . . . and to developing mutually beneficial and non-paternalistic partnerships with communities on behalf of individuals and defined populations” [8]. The skills needed to appropriately use an interpreter, navigate sensitive topics with an adolescent population, and provide high-quality care to diverse patients can be built when pediatricians actively practice cultural humility [9]. To build skills in these areas, we developed a formative standardized patient encounter (SPE) focusing on training pediatric residents to perform an adolescent interview with a non-English speaking standardized patient (SP) using a medical interpreter, emphasizing the importance and expectations of upholding patient confidentiality, and building skills in self-awareness and cultural humility. While others have developed simulated cases focusing on non-English speaking patients, no other simulation has focused on the importance of cultural humility while seeing adolescent patients with unique healthcare needs [10].

## Materials and methods

Educational context

The concept for this teaching SPE was developed from a partnership between a refugee organization and members of our pediatric faculty. During a prior study on refugee perspectives on experiences with local healthcare providers, members of the community voiced concerns about the potential for private medical information to be shared with others by their interpreter [3]. Concerns regarding confidentiality were identified, with the community noting that some languages are only spoken within small closely knit communities in the area. While education about privacy laws was given to the community, our pediatric residency leadership identified this as an area to emphasize for our resident learners. As a result, our team identified the need to focus on medical confidentiality in this simulation. A simulated case was then developed by a group of pediatric educators, including multiple academic pediatricians, a board-certified adolescent physician, and a simulation expert.

This SPE was conducted on three separate days and took place in the simulation lab at The Simulation Center at Fairbanks Hall at the Indiana University School of Medicine over the course of five hours of protected educational time, during the annual required residency simulation curriculum. The SPE was one of eight simulations completed by the residents, with each simulation lasting approximately 20 minutes. Pediatric residents and residents in pediatric combined training programs, henceforth known as residents, participated in this simulation, held approximately nine months, 11 months, and 13 months after the start of their intern years. This specific scenario was performed in a simulation room that was designed to imitate a clinic room setting. For completion of the SPE, we had a chair for the SP, an earpiece microphone for the SP, an exam table, a chair for the resident, a chair for the interpreter, and a one-way glass mirror.

Ethics approval and consent to participate

The need for informed consent and approval was waived by the ethics committee/Institutional Review Board of Indiana University as it was considered a quality improvement study. All methods were performed in accordance with the relevant guidelines and regulations of the Declaration of Helsinki.

Logistics

Residents rotated through the eight simulation scenarios in groups of three to five residents per group. Residents were randomly pre-assigned into groups by a simulation educator who had no prior knowledge of the communication skills or relevant background or experiences of the residents. The groups rotated the lead on each new scenario they worked through during their day at the simulation lab. With each participating group in this specific scenario, the lead resident went into the simulation room while the rest of the residents in the group were led by the facilitator to observe the SPE from behind a one-way mirror. The actors portraying the adolescent and the medical interpreter in the SPE were recruited by our team and were trained on how to act and react in the presence of the resident. Both actors were bilingual. For the first iteration, the simulation actors were bilingual in Spanish and English. For the second and third iterations, the simulation actors were bilingual in Hakha-Chin and English. An experienced pediatrician faculty member facilitated the SPE. The facilitator was able to instruct via an earpiece microphone to the SP about how to respond as needed.

**Figure 1:**
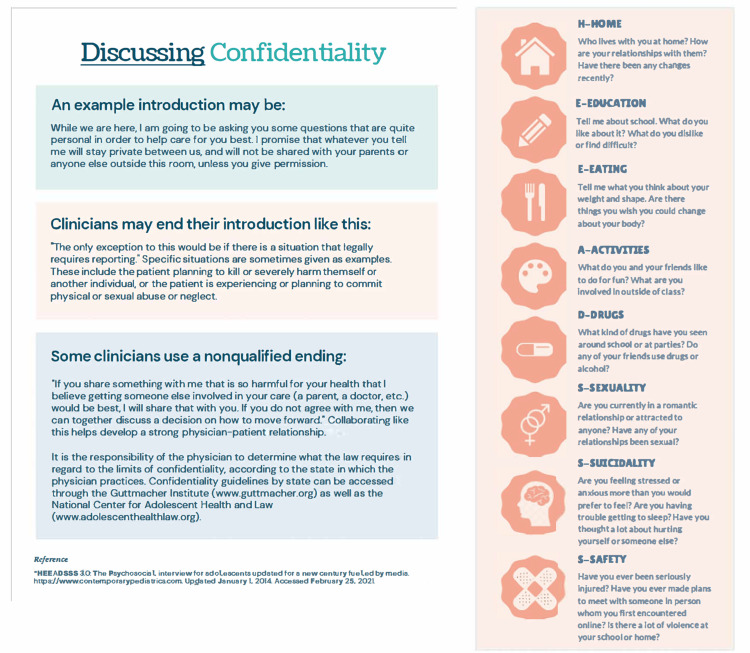
Fast Facts Information sheet (page 1) discussing the adolescent interview and confidentiality tips. Image Credit: Tristin Hudson, first author (image created using Canva, an online tool with templates available to create infographics by inputting text.)

**Figure 2:**
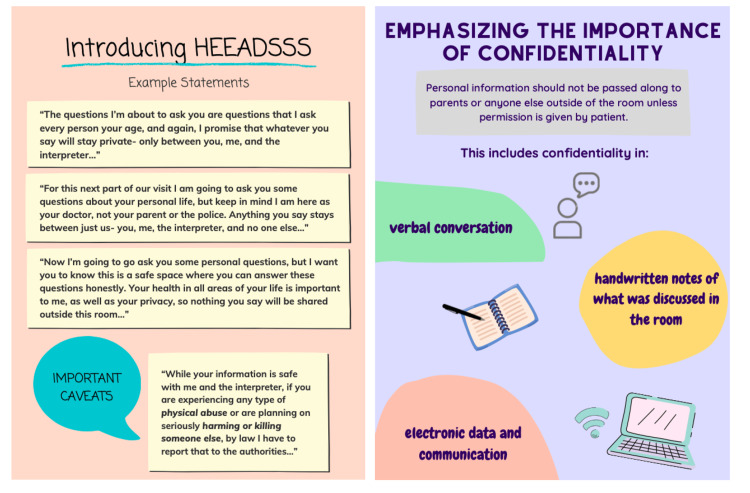
Fast Facts Information sheet (page 2) discussing the adolescent interview and confidentiality tips. Image Credit: Tristin Hudson, first author (image created using Canva, an online tool with templates available to create infographics by inputting text.)

For this simulation, the patient-facing scenario lasted approximately 10 minutes and was followed by a 10-minute debriefing with the group of residents. Before the resident entered the simulation room, they were informed that the patient was a 15-year-old non-English speaking female adolescent coming alone for a well-child check and that she needed an interpreter, who were both already seated in the room (see Appendix 1, “Simulation Case Template”(Tables 4-6)). The resident was to perform a HEEADSSS assessment which included sensitive health topics such as substance use, mental health, and sexual health. When questions that would be considered sensitive were posed, the SP would use body language to indicate that she was uncomfortable answering such questions about her health, which should prompt the resident to ask questions about her discomfort. The SP would then share her concerns regarding confidentiality. This response was designed to lead the resident into a discussion with the patient and interpreter about the importance of upholding medical confidentiality. After the resident completed the adolescent interview, the SPE was ended and the observing residents would join the individuals within the simulation room for the debriefing portion where all residents received information sheets discussing the adolescent interview and medical confidentiality (see Appendix 2, “Fast Facts,” which was created using Canva, an online tool with templates available to create infographics by inputting text (Figure 1 and Figure 2)).

Debriefing

Once the SPE was completed, there was a 10-minute debriefing period, in which there was feedback discussion among the residents, facilitator, interpreter, and SP. This debriefing discussion included a review of key learning objectives, such as conducting an adolescent interview using non-judgmental language, appropriately using an interpreter, being perceptive of the patient’s demeanor changes, and addressing concerns in an open-ended way that demonstrates cultural humility. There were also opportunities for the SP and interpreter as well as the residents to share their perspectives and thoughts on the session. The SP and interpreter were also able to offer feedback on how the resident navigated the case to further meet the objectives of the SPE and to develop skills with patients in the future.

Learner assessment

During the SPE, the observing residents were given a score sheet to evaluate the interaction between the actors and the lead resident, guided by the main objectives of the SPE (see Appendix 3, “Judges’ Scoring Sheet”(Table 7)). These evaluations were not included in the data analysis but were a tool to further engage the observing residents with the SPE and debriefing. Upon completion of the debriefing, all residents received a survey to reflect on their comfort before and after this SPE (see Appendix 4, “Post-Simulation Survey” (Table 8)). The survey comprised 10 questions exploring their comfort with adolescent patients, interpreter use, and diverse patient populations, with additional questions regarding skill building and a section for any additional comments. The survey questions and responses were selected by the authors from a validated self-reflection tool developed for an adolescent medicine rotation [11]. Each answer option was scored on a six-point Likert scale, anchored to degrees of agreement, enthusiasm, or comfort, depending on the aim of the question. The survey also asked for the resident’s name to collect characteristics data.

Analysis

The characteristics of the residents and the results of the survey were analyzed using descriptive statistics. Paired t-tests were performed to compare the results of the pre- and post-simulation questions. P-value (α) was set at 0.05. Unanswered survey questions were excluded from the analysis.

## Results

Of the 139 residents in their respective residency classes, 90 were present for the simulation day. Forty-nine of the residents did not attend any part of the simulation day, due to having clinical responsibilities outside of the pediatrics department or vacation time. One resident declined to participate in the research. The remaining 89 residents represented seven different residency programs (categorical pediatrics, internal medicine/pediatrics, emergency medicine/pediatrics, pediatrics/psychiatry/child psychiatry, neurodevelopmental disabilities, pediatrics-medical genetics, and child neurology). The characteristics of the participants are displayed in Table 1. All 89 residents responded to at least one question on the survey, with 84 residents answering all the questions.

**Table 1:** Characteristics of Resident Participants *Percentages may not add up to 100% due to rounding n, number; PGY, post-graduate year

Characteristic	Number of Participants n (%*), N=89
Gender	
Male	26 (29)
Female	60 (67)
Unknown	3 (3)
Post Graduate Year (PGY)	
PGY-1	66 (74)
PGY-2	23 (26)
Program	
Pediatrics	54 (61)
Internal Medicine/Pediatrics	15 (17)
Emergency Medicine/Pediatrics	2 (2)
Pediatrics/Psychiatry/Child Psychiatry	5 (6)
Neurodevelopmental Disabilities	3 (3)
Child Neurology	6 (7)
Pediatrics-Medical Genetics	1 (1)
Unknown	3 (3)

During the SPE, all lead residents were able to transition into aspects of the HEEADSSS interview and recognized the adolescent’s discomfort regarding their discussion of sensitive information. Within the survey, the mean comfort level between pre-simulation and post-simulation significantly increased (all with p<0.01) (Table 2).

**Table 2:** Results of the Post-simulation Survey Scores based on a six-point Likert scale, with 1=strongly disagree/very uncomfortable and 6=strongly agree/very comfortable

Item	Mean (Standard Deviation)		P-value
	Pre-simulation	Post-simulation	
On an enthusiasm scale, how would you generally rate your reaction to an adolescent walking in the door to see you?	3.67 (1.38)	4.09 (1.21)	<0.01
Rate what you perceive to be your level of comfort in the following scenarios. For the following questions, we define “comfort” as the ease with which you intellectually, emotionally, and physically feel about doing something.			
Communicating and gaining rapport with the adolescent	4.20 (0.89)	4.56 (0.80)	<0.01
Communicating with the appropriate use of an interpreter	4.71 (0.96)	5.05 (0.77)	<0.01
Discussing confidentiality with a patient	4.53 (1.09)	4.99 (0.87)	<0.01
Taking a sexual health history from an adolescent	3.86 (1.18)	4.44 (1.03)	<0.01
Exploring symptoms of depression in an adolescent	4.30 (0.93)	4.54 (0.85)	<0.01
Exploring alcohol and drug issues with an adolescent	4.38 (0.97)	4.62 (0.91)	<0.01
Engage in difficult conversations with diverse patient populations	4.07 (0.99)	4.52 (0.85)	<0.01

The mean difference between the Likert scores pre- and post-simulation ranged from +0.24 to +0.57 for enthusiasm to see adolescent patients, communicating and gaining rapport with the adolescent, appropriate use of an interpreter, discussing confidentiality, exploring depression symptoms, and exploring alcohol and drug issues. The greatest gains in Likert scores were seen in comfort taking a sexual health history, discussing confidentiality, and engaging in difficult conversations with diverse patient populations, with increases of +0.57, +0.45, and +0.45, respectively.

The majority of residents (97%, N=86) noted some level of agreement that this simulation helped them build skills in understanding cultural humility (mean Likert score: 4.91, SD: 0.87) as well as valuable skills to improve the care of future patients (mean: 5.10, SD: 0.87).

At the end of the survey, there was an opportunity to write comments about the simulation in a free-text section. Many residents left comments, often describing how they valued the simulation, though two comments did note that it would be helpful if all residents were able to be the lead residents (Table 3). 

**Table 3:** Examples of Post-simulation Survey Comments

Examples of comments
- “I think this was a good refresher! I run into this scenario all the time in clinic.”
- “Very useful reminder on how to use interpreter services. Good tips on HEEADSSS exam.”
- “I think this is a great exercise - I rarely get to practice having HEEADSSS exams conversations and simulation is a great way to practice.”
- “Difficult scenario but great opportunity to practice - appreciated the time to debrief.”
- “Discussing confidentiality is important but we also need to let them know that all prescriptions will show up on insurance so it’s also important to be open with their parents.”

## Discussion

The purpose of this SPE was to provide an opportunity for pediatric residents to navigate the challenges of communicating with non-English speaking adolescent patients in a way that fosters a trusting patient-provider relationship. The simulation’s focus was to conduct an adolescent interview using a medical interpreter and to discuss the importance of medical confidentiality. By addressing this issue in the SPE, residents were able to practice emphasizing the importance of upholding medical confidentiality with both the patient and the medical interpreter. Many of the residents agreed that the SPE helped them learn and build skills in cultural humility as well as improve care in future patient interactions.

While the combination of an adolescent interview and the use of an interpreter can be challenging, it is important as a recent study showed that adolescent refugees commonly have health concerns that require intervention [12]. Residents are taught how to conduct an adolescent interview and are aware of the importance of upholding the confidentiality of all patients. However, there are few opportunities to practice these skills within a safe and supportive environment. During the debriefing period of this SPE, many residents reported verbally or in writing that this session was helpful, as they seldom get to practice performing these exams. The residents also were reminded of appropriate communication using medical interpreters, such as maintaining eye contact with patients, speaking directly to them in short phrases, and allowing long enough time in pauses for the interpreter to communicate information to the patient [13].

The contribution of perspectives by the local Burmese refugee population of Indianapolis was a huge asset in creating a relevant and realistic scenario to improve the care of refugee and minority populations. In small and close-knit communities, medical interpreters may be known by the patients they work with, which could make having sensitive discussions more uncomfortable for the patient ­­[3,14]. If this concern is not addressed, patients may fail to receive quality care equitable to that which is received by English-speaking patients, as they may not feel comfortable sharing sensitive medical information or asking important questions. Receiving insights into specific challenges faced by immigrant and refugee patients in their medical visits allowed us to develop specific training objectives so that physicians can more thoughtfully engage with their future patients. By addressing concerns that local immigrant communities have voiced by educating physicians in training, our goal is to improve the relevant health inequities that these populations face.

There are a few limitations to the study. There were a small number of residents who were the lead residents of the SPE compared to the observing residents, and we were unable to compare the survey results of the lead resident to the results of the observing residents, though there is evidence that vicarious learning can be as effective as learning by doing [15]. Additionally, this specific SPE was only run at a single institution, therefore limiting its generalizability, though it was run three times with many learners overall and with two different languages. Finally, the retrospective pre- and post-survey design may have introduced recall bias, though was designed to avoid overconfidence bias. Other institutions may use this simulation but may need to modify its delivery if they do not have the technology to perform this simulation as described. For example, if an earpiece microphone for the SP is not available, the SP could be more extensively trained to avoid the need for real-time feedback. Additionally, various modifications may be made in the future to introduce different complexities of the case. For example, instead of a young woman as the interpreter, the scenario dynamics could be shifted significantly by introducing an older male interpreter for the 15-year-old female patient. We look forward to continuing to evolve the case in future administrations.

## Conclusions

Overall, 97% of residents agreed that this simulation helped them build skills in understanding cultural humility and improve the care of future patients. In addition, all residents experienced an increase in comfort in caring for non-English speaking adolescent patients, working with medical interpreters, and conducting both an adolescent interview and a discussion on medical confidentiality. Communication is a critical component of a patient-provider relationship and being intentional in developing trusting relationships with patients helps to improve the overall quality of healthcare they receive.

## Appendices

Appendix 1: simulation case template

**Table 4:** Simulation Objectives, Critical Actions, and Report Learner Receives Prior to the Simulation Before the resident entered the simulation room, they were informed that the patient was a 15-year-old non-English speaking female adolescent coming alone for a well-child check and that she needed an interpreter, who were both already seated in the room.

Simulation Case Template	
PATIENT NAME: Maria Lopez, 15 years old; CHIEF COMPLAINT: Well Child Check; PHYSICAL SETTING: Outpatient Clinic	
Brief narrative description of the case	A 15-year-old Mexican female is coming in alone, with permission from her mother, for a well-child check. An interpreter is needed for her visit. The resident is to conduct an adolescent interview, during which it is discovered that the patient is very uncomfortable discussing sensitive health information, especially sexual health, in the presence of a medical interpreter. The goal of this simulation case is for pediatric residents to practice conducting an adolescent interview (HEEADSSS exam) through the appropriate use of an interpreter, while also discussing the importance of upholding patient confidentiality.
Primary Learning Objectives	Describe the appropriate use of a medical interpreter for non-English speaking patients within the context of patient-centered care Demonstrate skills in adolescent interviewing Summarize the importance and expectations of upholding patient confidentiality Demonstrate social skills including self-awareness of cultural humility
Critical Actions	The goal is to have the resident conduct an adolescent interview (HEEADSSS exam) through appropriate use of an interpreter and also discuss the importance of upholding patient confidentiality. The resident should at some point in the adolescent interview ask questions regarding the sexual health of the standardized patient (SP), which should then prompt the SP to show discomfort and describe the “Back Story Concerns Regarding Confidentiality” (described below, a story about an adolescent in her community having her private health information become a topic of local gossip). The resident should engage the SP about confidentiality and work to understand her point of view. If done appropriately, the SP would be willing to discuss her sexual health in order to reduce risks of unplanned pregnancy, STIs, and other safety concerns.
Learner Preparation or Pre-work	Report learner receives prior to the simulation of a 15-year-old Mexican female adolescent coming alone for her well-child check today (The mother’s consent was provided for the patient to be seen alone today.) The patient speaks Spanish and said an interpreter was needed. Both the medical assistant and medical student confirm that an interpreter is needed. The trusted and thorough medical student saw the patient first. The patient has no significant medical history, no major surgeries, no hospitalizations, and no medications. The patient’s BMI is 22. A physical exam is completely unremarkable. She has stressors but screens negative for depression. She has never been tested for STIs. She has minimal exposure to drugs and violence.

**Table 5:** Backstory for the Simulated Patient Presented here are the vital signs and an example of a backstory for the simulated patient, in order to answer questions consistently between encounters.

Initial Presentation			
Initial Vital Signs	BP: 116/73 HR: 75 RR: 15 SpO2: 99 Temp: 97		
Overall Setting and Appearance	Outpatient clinic setting, a Mexican adolescent female patient and a medical interpreter both sitting in chairs next to the exam table		
Standardized Participants (and their roles in the room at case start)	Backstory for Latina actress: You’ve lived in the United States and (insert your specific local community information as applicable) for the last year. Our goal is to have the resident address the hesitancy of the actress to answer sensitive questions (sexual health/orientation, substance abuse, etc.) with the use of an interpreter.		
HPI	Be sure to answer promptly and comfortably for the initial questions that are non-sensitive. Start to show hesitancy when sensitive questions about drug/alcohol use are asked, perhaps by answering more shortly and looking at the floor. When sexual health questions are asked, there should be a noticeable demeanor change, such as being much slower to answer, or even just falling silent, and looking at both the doctor and the interpreter nervously. When the resident begins addressing your discomfort, say you are concerned about answering private questions with another person present who is not the doctor. Allow the resident to ask why that might be the case. No known COVID-19 symptoms or exposures if asked (if the simulation takes place during the COVID-19 pandemic)		
Past Medical/Surgical History	Medications	Allergies	Social/Family History
No significant medical history, no major surgeries	None	None	You grew up in a rural part of Mexico. You have not yet learned English fluently since immigrating here with your family. You live with your parents and your two younger siblings in a two-bedroom home. You have no symptoms of anything, except you don’t sleep well. Because both of your parents work long hours, you take on the responsibility of caring for your younger siblings most evenings. You are actively involved at your local church. When you first started school in the United States, you found it hard to make friends. Your boyfriend of six months now was the first person to openly engage with you at school and you two are currently in a close and trusting relationship. You do not smoke but you tried alcohol once at a party. You do not currently worry about money or getting enough food. No one in your family, including you, smokes, drinks, or does drugs of any kind. You have only been sexually active with your boyfriend, and you do not use birth control or condoms. You have not been sick or had any fevers/rashes.
Physical Examination: Normal physical exam findings			

**Table 6:** Instructor Notes Presented here is an ideal scenario flow of the simulated patient encounter.

Instructor Notes - Changes and CASE Branch Points		
Intervention/Time point	SP Script	Case Progression
Introduction (0-2 minutes)	Answer questions asked by the resident.	Warm engagement. Looks directly at the patient. Smiles. Introduces self. Assesses if the patient feels comfortable with an interpreter.
SP Concerns (2-6 minutes)	Introduction of community account of teen’s poor experience with a medical interpreter.	Support adolescents in this difficult event. The resident asking about what the adolescent thinks of this situation, and what the community thinks.
Discussion of Patient Confidentiality & Sexual Health (6-9 minutes)	Discussion of adolescent’s sexual health and other sensitive questions.	Ensures confidentiality of her answers and encourages open discussion of these topics.
10 minutes	Transition to another topic.	End scenario.

Ideal Scenario Flow

Our goal is to have the resident address the hesitancy of the SP to answer sensitive questions (sexual health/orientation, substance abuse, etc.) with the use of an interpreter.

Non-sensitive question topics may include safety at home, body image, school, etc.

Sensitive question topics include: drug/alcohol use (partially), sexual history (especially)

Here is an example of a way this could come up:

Be sure to answer promptly and comfortably for the initial questions that are non-sensitive. Start to show hesitancy when sensitive questions about drug/alcohol use are asked, perhaps by answering more shortly and looking at the floor. When sexual health questions are asked, there should be a noticeable demeanor change, such as being much slower to answer, or even just falling silent, and looking at both the doctor and the interpreter nervously 

We want to get the resident to ask the right questions: if the patient is uncomfortable, why that may be, what they can do to help, etc.

When the resident begins addressing your discomfort, you can look at the interpreter and say you’re unsure about answering such personal questions. Say you are concerned about answering private questions with another person present who is not the doctor. Allow for the resident to ask why that might be the case. Then go into the “Back Story Concerns Regarding Confidentiality” below.

Back story concerns regarding confidentiality: A friend of yours in the local community asked her doctor about birth control but needed to use a medical interpreter to do so. Soon after that appointment, there was gossip around the community about the teen being sexually active, although she had not been. The only discussion she had about it was between her and the doctor. She believes that it is possible her medical interpreter had disclosed this private information, as she knew the interpreter peripherally in the community.

You do not want any of the sensitive and private information about your personal health to be shared by an interpreter who may know people in your close-knit community. If the resident reassures you, however, that the information you share will not leave the room, you will be more willing to have these important conversations.

There are two anticipated management mistakes. One could be a failure to recognize the discomfort of the patient: some residents did not immediately recognize that the patient was becoming more and more uncomfortable, but instead took much longer to realize. This would initially lead to minimal sharing of important health information. We found it helpful to more clearly indicate discomfort by having the patient just stop answering questions altogether, specifically when it came to ones regarding sexual health. This would then lead the residents to address the hesitancy of the SP. Another could be difficulty in conducting the discussion regarding sexual health: We found when using this case with residents that some of them did not investigate with open-ended questions. During the debriefing that followed each session, we highlighted the value of nonjudgmental language in the way questions are framed during an adolescent interview.

Appendix 2: fast facts 

Appendix 3: judges’ scoring sheet

**Table 7:** Judges’ Scoring Sheet During the simulated patient encounter, the observing residents were given this score sheet to evaluate the interaction between the actors and the lead resident, guided by the main objectives of the simulated patient encounter.

Team Names:				
Judge:				
Positive, clear communication in the presence of an interpreter - Introduces self; speaks directly to patient; smiling at the patient, keeping eye contact with patient, speaking slowly, engages in rapport building				
1: Delayed or incorrect performance of most criteria	2: Delayed or incorrect performance of many criteria	3: Delayed or complete performance of some criteria	4: Competent performance of most criteria	5: Efficient and rapid performance of all criteria
Performs an adolescent interview - Asks questions related to the HEEADSSS questionnaire: home, education, eating, activities, drugs, sexuality, suicidality, and safety				
1: Delayed or incorrect performance of most criteria	2: Delayed or incorrect performance of many criteria	3: Delayed or complete performance of some criteria	4: Competent performance of most criteria	5: Efficient and rapid performance of all criteria
Probes for reason behind hesitancy and discomfort of patient - Asks open-ended questions about patient’s concerns, demonstrates awareness of patient’s demeanor change				
1: Delayed or incorrect performance of most criteria	2: Delayed or incorrect performance of many criteria	3: Delayed or complete performance of some criteria	4: Competent performance of most criteria	5: Efficient and rapid performance of all criteria
Explores adolescent’s beliefs with cultural humility – Asks about how the story made her feel, empathize with her, asks about her thoughts (and/or community’s stance) on confidentiality in a non-threatening or non-accusatory manner				
1: Delayed or incorrect performance of most criteria	2: Delayed or incorrect performance of many criteria	3: Delayed or complete performance of some criteria	4: Competent performance of most criteria	5: Efficient and rapid performance of all criteria
Engages in supportive interactions around the topic of confidentiality – Acknowledges the importance of adolescent’s knowledge/feelings, expresses shared goals for adolescent				
1: Delayed or incorrect performance of most criteria	2: Delayed or incorrect performance of many criteria	3: Delayed or complete performance of some criteria	4: Competent performance of most criteria	5: Efficient and rapid performance of all criteria
Additional Comments:				

Appendix 4: post-simulation survey

**Table 8:** Post-Simulation Survey Upon completion of the debriefing, all residents received a survey to reflect on their comfort before and after this simulated patient encounter.

Post-simulation Survey						
This scenario is part of an educational research study. We are asking for you to participate in this research study, which will involve completing this survey, engaging with the simulation scenarios and debriefing, and completing another survey at the end. Your participation is voluntary, and if you are not interested in having your results included, please check the box below (next to your name) and return the blank survey. There are no risks of participating in this research. If you have any questions about this research, please contact Megan McHenry, MD, MS at .						
Name:						
Opt-out of research □						
Please mark the response that describes your stance on the following statements.						
On an enthusiasm scale, how would you generally rate your reaction to an adolescent walking in the door to see you?						
Pre-simulation	1 Very Unenthusiastic	2 Moderately Unenthusiastic	3 Slightly Unenthusiastic	4 Slightly Enthusiastic	5 Moderately Enthusiastic	6 Strongly Enthusiastic
Post-simulation	1 Very Unenthusiastic	2 Moderately Unenthusiastic	3 Slightly Unenthusiastic	4 Slightly Enthusiastic	5 Moderately Enthusiastic	6 Strongly Enthusiastic

Rate what you perceive to be your level of comfort in the following scenarios. For the following questions, we define “comfort” as the ease with which you intellectually, emotionally, and physically feel about doing something.						
Communicating and gaining rapport with the adolescent						
Pre-simulation	1 Very Uncomfortable	2 Moderately Uncomfortable	3 Slightly Uncomfortable	4 Slightly Comfortable	5 Moderately Comfortable	6 Strongly Comfortable
Post-simulation	1 Very Uncomfortable	2 Moderately Uncomfortable	3 Slightly Uncomfortable	4 Slightly Comfortable	5 Moderately Comfortable	6 Strongly Comfortable

Communicating with the appropriate use of an interpreter						
Pre-simulation	1 Very Uncomfortable	2 Moderately Uncomfortable	3 Slightly Uncomfortable	4 Slightly Comfortable	5 Moderately Comfortable	6 Strongly Comfortable
Post-simulation	1 Very Uncomfortable	2 Moderately Uncomfortable	3 Slightly Uncomfortable	4 Slightly Comfortable	5 Moderately Comfortable	6 Strongly Comfortable

Discussing confidentiality with a patient						
Pre-simulation	1 Very Uncomfortable	2 Moderately Uncomfortable	3 Slightly Uncomfortable	4 Slightly Comfortable	5 Moderately Comfortable	6 Strongly Comfortable
Post-simulation	1 Very Uncomfortable	2 Moderately Uncomfortable	3 Slightly Uncomfortable	4 Slightly Comfortable	5 Moderately Comfortable	6 Strongly Comfortable

Taking a sexual health history from an adolescent						
Pre-simulation	1 Very Uncomfortable	2 Moderately Uncomfortable	3 Slightly Uncomfortable	4 Slightly Comfortable	5 Moderately Comfortable	6 Strongly Comfortable
Post-simulation	1 Very Uncomfortable	2 Moderately Uncomfortable	3 Slightly Uncomfortable	4 Slightly Comfortable	5 Moderately Comfortable	6 Strongly Comfortable

Exploring symptoms of depression in an adolescent						
Pre-simulation	1 Very Uncomfortable	2 Moderately Uncomfortable	3 Slightly Uncomfortable	4 Slightly Comfortable	5 Moderately Comfortable	6 Strongly Comfortable
Post-simulation	1 Very Uncomfortable	2 Moderately Uncomfortable	3 Slightly Uncomfortable	4 Slightly Comfortable	5 Moderately Comfortable	6 Strongly Comfortable

Exploring alcohol and drug issues with an adolescent						
Pre-simulation	1 Very Uncomfortable	2 Moderately Uncomfortable	3 Slightly Uncomfortable	4 Slightly Comfortable	5 Moderately Comfortable	6 Strongly Comfortable
Post-simulation	1 Very Uncomfortable	2 Moderately Uncomfortable	3 Slightly Uncomfortable	4 Slightly Comfortable	5 Moderately Comfortable	6 Strongly Comfortable

Engage in difficult conversations with diverse patient populations						
Pre-simulation	1 Very Uncomfortable	2 Moderately Uncomfortable	3 Slightly Uncomfortable	4 Slightly Comfortable	5 Moderately Comfortable	6 Strongly Comfortable
Post-simulation	1 Very Uncomfortable	2 Moderately Uncomfortable	3 Slightly Uncomfortable	4 Slightly Comfortable	5 Moderately Comfortable	6 Strongly Comfortable

This scenario helped me build skills in understanding cultural humility.						
Post-simulation	1 Strongly Disagree	2 Moderately Disagree	3 Slightly Disagree	4 Slightly Agree	5 Moderately Agree	6 Strongly Agree

I learned valuable skills to improve the care of my future patients because of this scenario.						
Post-simulation	1 Strongly Disagree	2 Moderately Disagree	3 Slightly Disagree	4 Slightly Agree	5 Moderately Agree	6 Strongly Agree
Please provide any additional comments you may have about today’s scenario:						
